# The AP-1 Sigma Subunit Gene *PsAP1* Acts as a Key Pathogenicity Factor by Regulating Metabolic Reprogramming in *Puccinia striiformis* f. sp. *tritici*

**DOI:** 10.3390/jof12010057

**Published:** 2026-01-12

**Authors:** Beibei Liu, Jianing Wu, Guoshuai Zhang, Jianghua Chen, Guangkuo Li, Xintong Wang, W. G. Dilantha Fernando, Haifeng Gao, Yue Li

**Affiliations:** 1 Key Laboratory of Biological Ecological Adaptation and Evolution in Extreme Environments, College of Life Science, Xinjiang Agricultural University, Urumqi 830052, China; bi916228271@163.com (B.L.); 17325221793@163.com (G.Z.); 2 Institute of Plant Protection, Xinjiang Academy of Agricultural Sciences/Key Laboratory of Integrated Pest Management on Crop in Northwestern Oasis, Ministry of Agriculture and Rural Affairs, Urumqi 830091, China; jianingwu@webmail.hzau.edu.cn (J.W.); jianghuachen2024@126.com (J.C.); lgk990808@163.com (G.L.); xintongwang@nwafu.edu.cn (X.W.); ghf20044666@163.com (H.G.); 3 Department of Plant Science, University of Manitoba, Winnipeg, MB CAR3T2N2, Canada; dilantha.fernando@umanitoba.ca

**Keywords:** wheat stripe rust, AP-1 complex, metabolic reprogramming, HIGS

## Abstract

Wheat stripe rust, caused by *Puccinia striiformis* f. sp. *tritici* (Pst), poses a severe threat to global wheat production. The adaptor protein complex AP-1 plays a crucial role in vesicular trafficking, yet its function in rust fungi remains poorly understood. In this study, a gene encoding an AP-1 σ subunit, designated PsAP1, was identified in Pst. The expression of PsAP1 was highly induced during the early infection stage. Heterologous expression of PsAP1 in a *Fusarium graminearum* mutant partially restored its pathogenic defects. Subcellular localization analysis revealed that PsAP1 localizes to the plasma membrane, cytoplasm, and nucleus. Silencing PsAP1 in wheat using Barley stripe mosaic virus-mediated host-induced gene silencing (BSMV-HIGS) significantly attenuated Pst pathogenicity, reducing hyphal growth by 6.7% (colony diameter), sporulation by 61.6% (lesion length), and pathogen biomass by 66%, along with enhanced accumulation of host reactive oxygen species. Transcriptomic analysis further demonstrated that silencing PsAP1 disrupted multiple pathways, including MAPK signaling, glutathione metabolism, and carbohydrate metabolism. These findings indicate that PsAP1 facilitates Pst infection by modulating vesicular trafficking, suppressing host immunity, and reprogramming host metabolism. This study provides novel insights into the pathogenic mechanisms of rust fungi and suggests a potential target for disease control.

## 1. Introduction

Wheat stripe rust caused by *Puccinia striiformis* f. sp. *tritici* (Pst), represents a major biotic constraint to global wheat production. With less than 9% of global arable land, China accounts for approximately 25% of the world grain output, making protection against such diseases particularly crucial for food security [1]. This heteroecious pathogen requires both wheat and alternate hosts, such as *Berberis* spp., to complete its life cycle and can cause yield losses of up to 100% during severe epidemics [2,3].

Recent research has significantly advanced our understanding of the molecular mechanisms underlying Pst pathogenesis. Studies have revealed that Pst can hijack host sugar transporters via secreted effector proteins [4]. For instance, the effector protein Pst_12806 targets TaSWEET14d to compete for sucrose [5], while Pst_4,88 regulates the ABA pathway to activate TaSTP6 expression, thereby enhancing hexose acquisition [6]. Furthermore, effector proteins, such as HASP215, suppress host immunity by targeting the wheat MAPK signaling cascade (TaMKK2-TaMAPK6-TaSGT1) [7]. Recent findings also indicate that Pst utilizes the effector Pst9653 to interfere with the subcellular localization of the catalase TaCAT3, facilitating ROS scavenging during infection [8]. Additionally, the pathogen can employ Pst_TTP1 to inhibit chloroplast-mediated leaf senescence, thereby extending the window for nutrient acquisition [9]. Despite these advances, a comprehensive understanding of Pst pathogenicity remains challenging, primarily due to the lack of a robust genetic transformation system. Although biolistic methods have been explored for creating mutant libraries of Pst, their efficiency and applicability remain limited, directly impeding functional studies of key virulence genes [10].

Adaptor Protein Complex 1 (AP-1), a highly conserved heterotetramer composed of γ, β1, μ1, and σ1 subunits, serves as a central regulator of intracellular membrane trafficking. By recognizing specific sorting motifs ([DE]XXXL[LI] and YXXΦ), AP-1 mediates cargo protein selection and facilitates vesicle formation between the trans-Golgi network and endosomes [11,12]. Research across diverse organisms has demonstrated the essential nature of AP-1 components, with mutations causing embryonic lethality in mice [13,14], pollen defects in *Arabidopsis* [15,16,17], and growth impairment in fission yeast [18]. In the protozoan pathogen *Toxoplasma gondii*, AP1μ is required for host cell invasion [19]. Collectively, these findings suggest that AP-1-dependent membrane trafficking may play a conserved and critical role in microbial pathogenesis.

Our preliminary work identified *PsAP1* as encoding a putative AP-1 σ subunit in Pst that shares conserved domains with FgAP1 from *Fusarium graminearum* (*F. graminearum*) [20]. Given the central role of AP-1 complexes in coordinating membrane trafficking with cellular metabolic homeostasis and emerging evidence that various pathogens manipulate host nutrients and immune pathways to facilitate infection, we hypothesize that *PsAP1* may represent a previously uncharacterized virulence determinant in wheat stripe rust. We propose that *PsAP1* potentially regulates the metabolism and utilization of critical compounds such as nucleotides and thiamine through vesicle-mediated transport, thereby contributing to pathogen virulence. This study aimed to functionally characterize *PsAP1* using Host-Induced Gene Silencing (HIGS) and transcriptomic approaches [21,22,23,24], with the goal of revealing novel mechanisms of Pst pathogenesis and identifying potential targets for sustainable control strategies.

## 2. Materials and Methods

### 2.1. Plant and Fungal Materials

The wheat cultivar Mingxian 169 was primarily used for the propagation of the wheat stripe rust pathogen physiological race CYR32, whereas Shuiyuan 11 was employed for gene expression profiling and HIGS experiments. All experimental materials mentioned above were provided by our laboratory. Seeds were surface-sterilized by immersion in 75% ethanol for 30 s, rinsed thoroughly, and then soaked overnight until germination. Germinated seeds were placed ventral groove-down on 6 g/L water agar in 90 mm Petri dishes, which were covered with plastic film to maintain humidity and incubated at 25 °C in darkness for 2 days. Plants were grown in square pots (7 × 7 × 7 cm) filled to two-thirds of their volume with a mixed substrate (charcoal–perlite–vermiculite–nutrient soil, 1:0.5:1:2, *v*/*v*/*v*/*v*). Growth conditions were maintained at 15 °C under a 16 h light/8 h dark photoperiod.

Wheat stripe rust was maintained on the wheat plants. At the one-leaf-one-heart stage, wheat leaves were inoculated with a mixture of urediniospores and Electron^®^ fluorinated liquid. Newly formed urediniospores were collected in clean tubes after 14 d. *Nicotiana benthamiana* (*N. benthamiana*), physiological race CYR32, and the vector pYBA1132 were provided by Institute of Plant Protection, Xinjiang Uygur Autonomous Region Academy of Agricultural SciencesXinjiang/Key Laboratory of Integrated Pest Management on Crop in Northwestern Oasis, Ministry of Agriculture and Rural Affairs. The BSMV:α, BSMV:β, BSMV:γ, and BSMV:PDS vectors were kindly supplied by the research group of Prof. Xiaojie Wang at Northwest A&F University.

### 2.2. Cloning and Bioinformatic Analysis of PsAP1

One-leaf, one-heart stage wheat leaves were immediately frozen in liquid nitrogen. The samples were ground to a homogeneous powder using a fully automated rapid grinder (60 Hz, 3 min). Total RNA was extracted using the Polysaccharide and Polyphenol Plant Total RNA Extraction Kit (Tiangen, Beijing, China), and cDNA was synthesized using a reverse transcription kit (Vazyme, Nanjing, China). The full-length sequence of PsAP1 (XM_047955384.1) was downloaded from the NCBI website (https://www.ncbi.nlm.nih.gov/), and gene-specific primers for amplifying the coding sequence (CDS) region of PsAP1 were designed using Primer Premier software (version 6.0, Premier Biosoft, Palo Alto, CA, USA). Meanwhile, AP1 homologous protein sequences from Pst and other species were downloaded from the NCBI database. A phylogenetic tree was constructed using the MEGA11 software to analyze the evolutionary relationships of this protein among different species. Multiple sequence alignment of AP1 proteins from various species was performed using the ESPript 3.0 website (https://espript.ibcp.fr/ESPript/cgi-bin/ESPript.cgi (accessed on 1 October 2024)), and the conserved motifs of the AP1 protein in Pst were analyzed using the MEME website (https://meme-suite.org/meme/tools/meme (accessed on 1 October 2024)).

### 2.3. Expression Profiling of the PsAP1 Gene in Wheat Stripe Rust Fungus

Wheat plants at the ‘one-leaf-one-heart’ stage were inoculated with the stripe rust pathogen. Leaf samples were collected at 6, 12, 18, 24, 36, 48, 72, 120, 168, and 216 h post-inoculation (hpi). Gene-specific primers for qRT-PCR analysis of *PsAP1* expression were designed using Primer Premier software. The elongation factor gene (*PsEF*) [25] of Pst was used as an internal reference gene, and the relative expression level of *PsAP1* was calculated using the 2^−ΔΔCt^ method, with the urediniospore stage serving as the calibrator. Three biological and three technical replicates were performed for each sample.

### 2.4. Subcellular Localization of the PsAP1 Protein

Specific primers for PsAP1 were designed using Primer Premier software, and the fragment was inserted into the expression vector pYBA1132, which contains a green fluorescent protein (GFP) gene, using the ClonExpress^®^ II One Step Cloning Kit (Vazyme, Nanjing, China). The successfully constructed recombinant plasmid was transformed into *Agrobacterium tumefaciens* GV3101 and delivered to the lower epidermis of *N. benthamiana* leaves via *Agrobacterium*-mediated transient expression. At 48 hpi, subcellular localization of the PsAP1-GFP fusion protein was observed using an LSM900 (Zeiss, Jena, Germany) confocal laser scanning microscope.

### 2.5. Generation and Colony Morphological Characterization of Heterologous Knockout Mutants and Complementation Strains

To investigate whether *PsAP1* is involved in the pathogenicity of wheat stripe rust fungus (Pst), a gene-knockout approach was used in this study. Due to the lack of well-established mutant construction methods for Pst, a *F. graminearum FgAP1* (XP_011318961.1) mutant was generated to infer the function of *PsAP1*. Homologous fragments flanking the *FgAP1* gene were amplified using two rounds of PCR and fused with partial sequences of the hygromycin resistance gene. These two DNA fragments were co-transformed into *F. graminearum* protoplasts via PEG-mediated transformation, leading to the replacement of the *FgAP1* gene through homologous recombination and yielding putative knockout strains.

The complemented strain of *F. graminearum* was obtained using PEG-mediated protoplast transformation. A construct containing the native promoter of *FgAP1* (800 bp upstream of the start codon), the CDS of the target gene *PsAP1*, and the native terminator of *FgAP1* (800 bp downstream of the stop codon) was assembled into the pGTN plasmid. This recombinant plasmid was then transformed into protoplasts of the *F. graminearum* mutant strain, resulting in the generation of a complementation strain.

Mycelial plugs from wild-type (WT), mutant, and complementation strains cultured in SYM (Sucrose Yeast extract Medium) for 3 days were collected using a cork borer of uniform size. Fresh mycelial plugs of identical size were inoculated onto CM (Complete Medium) solid medium and each transformant was inoculated onto at least three plates. The plates were incubated at 28 °C for 5 days, after which the mycelial morphology was photographed and colony diameters were measured. Three biological replicates were used for each experiment.

### 2.6. Pathogenicity Assay of F. graminearum

Conidia were harvested from the CMC (Carboxymethylcellulose Sodium Medium) liquid medium and the supernatant was discarded. The conidial pellet was resuspended in sterile water, and the conidial concentration was adjusted to 1 × 10^6^/mL using a hemocytometer. When wheat coleoptiles reached approximately 1 cm in length, their tips were excised with scissors to create wounds. A 2 µL aliquot of the adjusted conidial suspension was inoculated onto each wound site. Inoculated coleoptiles were air-dried in a ventilated area. Petri dishes containing coleoptiles were placed in a foam box lined with two layers of moist blotting paper at the bottom. The box was maintained at high humidity by spraying with water, sealed at the top with plastic wrap, and incubated at 25 °C for 7 days. After incubation, the lesion lengths were measured and photographed.

### 2.7. Host-Induced Gene Silencing (HIGS)

A unique approximately 200 bp fragment of the rust fungus *PsAP1* was selected and inserted into the γ vector using seamless cloning technology. The constructed recombinant vector was transformed into *Escherichia coli*, and single colonies were picked for sequencing verification. Plasmids extracted from sequence-verified colonies were stored at −20 °C for subsequent use. The BSMV:α, BSMV:β, BSMV:γ, BSMV:PDS, and BSMV:PsAP1 vectors were linearized separately, followed by in vitro transcription using a commercial transcription kit to obtain RNA viruses.

When wheat cultivar Shuiyuan 11 (Su11) reached the one-leaf-one-heart stage, virus inoculation was performed using a pre-prepared Fes buffer (containing 1.0 g sodium pyrophosphate, 1.0 g diatomaceous earth, 0.75 g glycine, 1.0 g bentonite, and 1.05 g K_2_HPO_4_). The experimental design included Fes buffer as the blank control, BSMV:PDS as the positive control, and BSMV:γ as the negative control. Ten days after virus inoculation, the treated leaves were challenge-inoculated with wheat stripe rust fungus. Disease phenotypes were observed at 14 dpi. Leaf samples were collected at 24, 48, and 72 hpi after rust inoculation to assess gene silencing efficiency and for histological analysis.

### 2.8. Histological Assays of Fungal Structures

Tissue samples were stained with hydrochloric acid solution (pH 3.8) containing 0.1% 3,3′-diaminobenzidine (DAB) to detect reactive oxygen species (ROS). Following ROS staining, samples were stained with wheat germ agglutinin (WGA). Fungal structures, including substomatal vesicles, infection hyphae, and haustorial mother cells, were examined using a ZEISS LSM 900 confocal laser scanning microscope (Jena, Germany). For each experimental group, a minimum of 50 infection sites were observed across three independent biological replicates. Hyphal length and area were quantified using ImageJ software (version 1.54, National Institutes of Health, Bethesda, MD, USA) and statistical analyses were performed using GraphPad Prism software (8.0.0, San Diego, CA, USA).

### 2.9. Transcriptome Library Construction

Leaf samples were collected at 24 and 48 hpi from both *PsAP1*-silenced experimental groups and the corresponding control groups, with three biological replicates per condition. All collected samples were immediately flash-frozen in liquid nitrogen and stored at −80 °C for subsequent analysis. A total of 12 samples were sent to Shanghai Aonasite Biotechnology Co., Ltd. for eukaryotic reference-based transcriptome sequencing. PolyA mRNA was isolated using magnetic beads (TruSeq RNA Sample Prep Kit, Illumina, San Diego, CA, USA). Double-stranded cDNA was synthesized and amplified, followed by size selection (200–500 bp) using 2% agarose gel electrophoresis (Bio-Rad, Hercules, CA, USA). Sequencing was performed on an Illumina HiSeq X Ten platform with a 2 × 150 bp paired-end read configuration.

Raw sequencing reads were processed to remove adapter sequences and low-quality reads (defined as those with >50% of bases with a quality score ≤ 10 or an N content exceeding 10%), yielding high-quality clean reads. All subsequent analyses were based on these clean reads. Alignment to the reference genome was conducted using HISAT2, and transcript assembly and quantification were performed using StringTie (8.0.0, San Diego, CA, USA) [26,27].

### 2.10. Analysis and Validation of Differentially Expressed Genes

Differentially expressed genes (DEGs) identified through screening were functionally annotated using the Gene Ontology (GO) database and pathway enrichment analysis via the Kyoto Encyclopedia of Genes and Genomes (KEGG). GO enrichment analysis was performed using ClusterProfiler software (4.0.0, Shenzhen, Guangdong, China), with an adjusted *p*-value (adj. *p*-value) < 0.05, set as the threshold for significant enrichment. KEGG pathway analysis was conducted on the KOBAS platform using a false discovery rate (FDR) < 0.05 as the significance cutoff. To validate the reliability of the transcriptome sequencing results, 15 DEGs selected based on KEGG pathway analysis were verified using quantitative real-time PCR (qRT-PCR). The elongation factor gene *PsEF* from wheat stripe rust fungus was used as an internal reference. Amplification was performed using the TB Green^®^ Premix Ex Taq™ II kit on a QuantStudio 6 Flex real-time PCR system. Three technical and three biological replicates were included for each sample. The relative expression levels of the genes were calculated using the 2^−ΔΔCt^ method, and the results were correlated with the RNA-seq data.

## 3. Results

### 3.1. Cloning and Bioinformatic Analysis of PsAP1

PCR amplification was performed using the cDNA derived from Pst-infected wheat (cv. Suiyuan 11) produced products ranging in size from 250 to 500 bp (Figure S1), which aligned with the anticipated size of the *PsAP1* coding sequence, thereby confirming the successful cloning of the gene.

**Figure 1:**
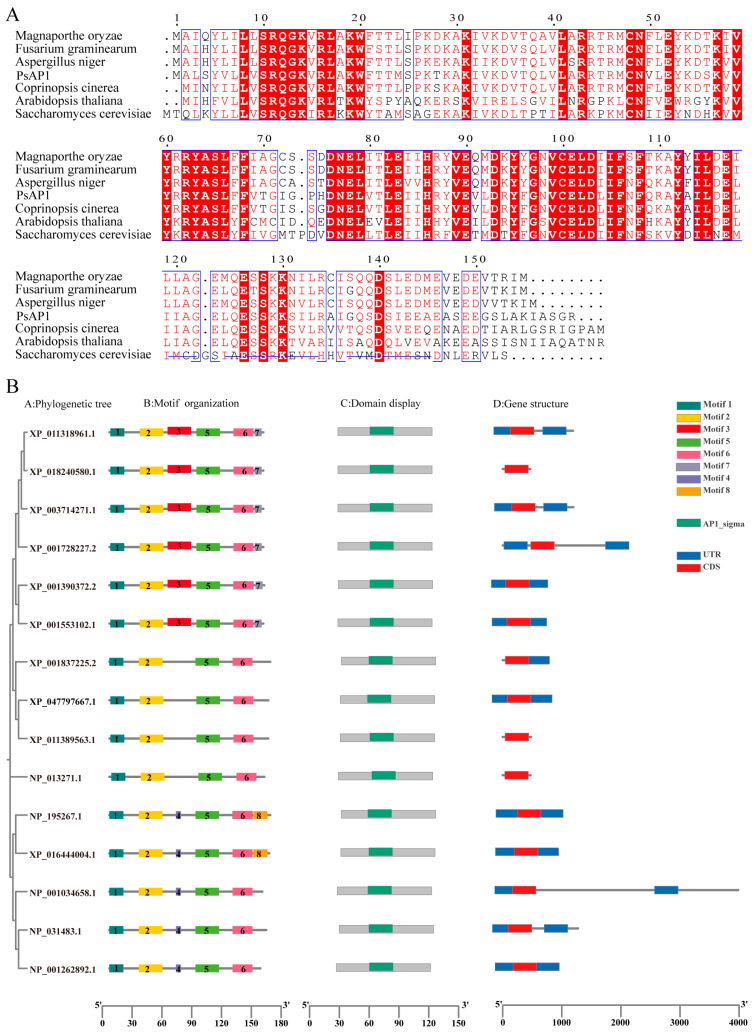
Gene structure and protein sequence characteristics of the PsAP1. (**A**) Multiple sequence alignment of AP1 protein from different species; White letters on a red square represent prevalent sequence residues, while red is used to depict analogous residues, blue boxes indicate conserved sequences. (**B**) Phylogenetic tree analysis. Gene structure and protein sequence characteristics of the PsAP1 protein in wheat stripe rust.

To clarify the evolutionary connections between various pathogenic fungi and microbes, an alignment of AP1 protein sequences was performed. The results of multiple sequence alignment indicated that PsAP1, similar to its homologs, possesses a conserved AP1_sigma domain (Figure 1A). Phylogenetic analysis using MEGA11 revealed that PsAP1 was most closely related to orthologs from *Ustilago maydis* and *Coprinopsis cinerea*. Additionally, MEME analysis identified eight conserved motifs across 15 AP1 proteins (Figure 1B), highlighting the significant conservation in both the composition and arrangement of motifs within the AP1 family.

### 3.2. Induction of PsAP1 by Pst Infection and Its Subcellular Localization to the Plasma Membrane and Nucleus

**Figure 2:**
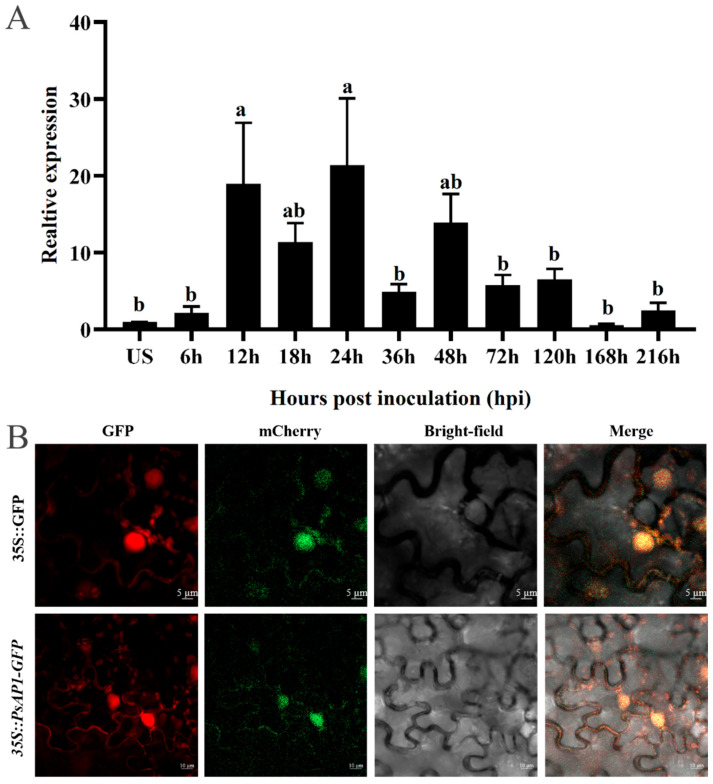
Expression Analysis and Subcellular Localization of *PsAP1*. (**A**) The transcriptional expression profile of the *PsAP1*. The same lowercase letters at the same time point indicate no significant difference (*p* > 0.05), whereas different lowercase letters at the same time point indicate significant differences (*p* < 0.05), with US standardized at 1. “US” is the abbreviation for Urediospores, which serve as the calibrator sample. (**B**) Subcellular localization of PsAP1.

To investigate the transcriptional expression profile of the *PsAP1* gene during the infection stages of Pst, we utilized quantitative real-time PCR (qRT-PCR) to measure its transcript levels. The transcription of *PsAP1* was found to increase, reaching its highest level during the early “parasitic/biotrophic” phase of infection. Specifically, there was an 18.94-fold increase in expression at 12 hpi with the Pst race CYR32, which corresponds to the substomatal vesicle formation stage. Expression peaked at 24 hpi, which is a crucial stage for haustorium formation. A second notable expression peak was observed at 48 hpi, corresponding to the period of active secondary hyphal growth and the establishment of a robust biotrophic interface with the host. Subsequently, transcript levels gradually decreased by 120 hpi (Figure 2A). These results suggested that *PsAP1* is significantly expressed during the initial stages of host infection, highlighting its potential role in the infectivity and pathogenicity of the pathogen.

To determine the subcellular localization of the protein encoded by *PsAP1*, we used *Agrobacterium*-mediated transient expression to express the pYBA1132:PsAP1-GFP fusion protein in tobacco leaf epidermal cells, with the empty pYBA1132-GFP vector serving as a control. Additionally, co-localization was performed using nuclear markers (red) and plasma membrane markers (red). As shown in the results, GFP fluorescence signals from the empty pYBA1132-GFP vector were uniformly distributed throughout the cytoplasm and nucleus of tobacco leaf epidermal cells. In contrast, GFP signals from the pYBA1132:PsAP1-GFP fusion construct were predominantly localized to both the plasma membrane and the nucleus. These signals showed a strong overlap with the red fluorescence from the plasma membrane marker and the nuclear marker, producing distinct yellow fluorescence in the merged channels (Figure 2B). This co-localization confirms that the protein encoded by the PsAP1 localizes to both the plasma membrane and the nucleus.

### 3.3. Mutation of FgAP1 Results in Delayed Hyphal Growth and Reduced Pathogenicity in F. graminearum

**Figure 3:**
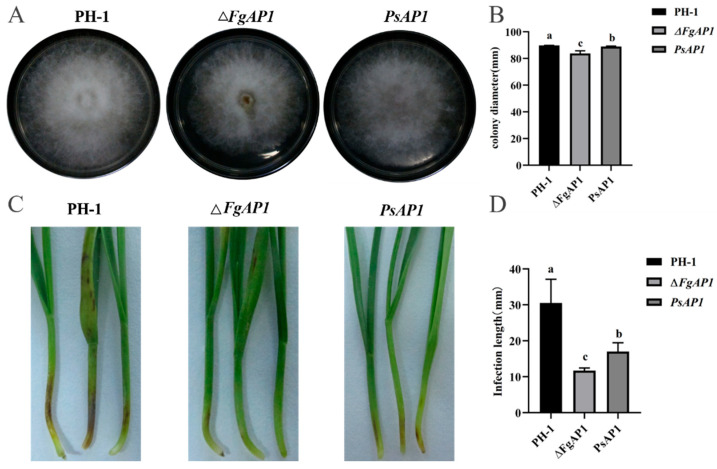
Heterologous complementation of *PsAP1* partially restores colony morphology and pathogenicity in *F. graminearum* FgAP1 mutant. (**A**) Colony morphology of the WT PH-1, ΔFgAP1 mutant, and PsAP1-complemented strains cultured on CM solid medium for 5 days. (**B**) Statistical analysis of colony diameters. Colony diameters were measured directly. The statistical significance of differences between the compared groups was determined using an unpaired two-tailed Student’s *t*-test performed with GraphPad Prism software. (**C**) Pathogenicity assay of wheat coleoptiles inoculated with conidia from the WT PH-1, ΔFgAP1 mutant, and PsAP1-complemented strains. (**D**) Statistical analysis of lesion length. Different lowercase letters indicate significant differences (*p* < 0.05). Three biological and three technical replicates were performed for each sample.

To evaluate the effect of FgAP1 deletion on hyphal growth, the FgAP1 knockout mutant (ΔFgAP1) and PsAP1-complemented strain were cultured on CM solid medium. The colony size of the ΔFgAP1 mutant was significantly smaller than that of the WT PH-1 strain (Figure 3A,B). Conversely, the PsAP1-complemented strain exhibited significantly larger colonies than the mutant strain, closely resembling the morphology of WT. The average colony diameters were 89.78 mm for the WT PH-1, 83.75 mm for ΔFgAP1, and 88.93 mm for the complemented strain, indicating that FgAP1 plays a role in regulating hyphal growth in *F. graminearum*.

To further explore the function of *PsAP1* in the context of *F. graminearum* infection, conidia from the WT PH-1, mutant ΔFgAP1, and complemented *PsAP1* strains were inoculated onto wheat coleoptiles. The findings revealed that the mutant strain showed a significant reduction in lesion extension from the inoculation site, with lesion lengths decreasing markedly from 30.50 mm in the WT to 11.70 mm. In contrast, the complemented strain exhibited a substantial increase in lesion length (17.00 mm) compared with the mutant strain. Consequently, *PsAP1* is integral to the pathogenicity of *F. graminearum* during coleoptile infection in wheat.

### 3.4. Transient Silencing of PsAP1 Reduces the Pathogenicity of Pst

**Figure 4:**
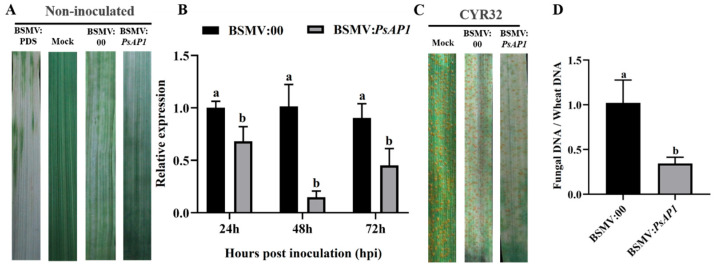
Transient silencing of *PsAP1* via BSMV-HIGS reduced the pathogenicity of Pst. (**A**) Leaf phenotypes at 10 days after virus inoculation. BSMV:PDS (positive control) shows photobleaching, BSMV:γ (negative control) shows abaxial yellowing, and BSMV:PsAP1 shows mosaic symptoms. (**B**) Silencing efficiency of *PsAP1* measured by qRT-PCR at 24, 48, and 72 hpi with Pst. Data are presented as the mean ± SD (*n* = 3). Different lowercase letters above the bars indicate statistically significant differences between the treatment and control groups at the same time point, as determined by one-way ANOVA followed by Tukey’s test (*p* < 0.05). (**C**) Disease phenotypes at 14 dpi with Pst race CYR32, showing reduced uredinia formation on BSMV:PsAP1-treated leaves compared to the control. (**D**) Relative pathogen biomass quantification in control and BSMV:PsAP1-treated leaves at 14 dpi with Pst. Different lowercase letters at the same time point indicate significant differences (*p* < 0.05).

To determine the function of *PsAP1* in Pst infection and pathogenicity, HIGS was performed using the Barley Stripe Mosaic Virus (BSMV) system. Ten days after silencing, the leaves treated with BSMV:PDS exhibited clear photobleaching, those treated with BSMV:γ showed yellowing on the abaxial surface, and BSMV:PsAP1-treated leaves presented mosaic symptoms (Figure 4A), indicating the successful establishment of a BSMV-mediated gene-silencing system. To assess silencing efficiency, qRT-PCR was conducted at 24, 48, and 72 hpi following Pst inoculation. The results demonstrated that *PsAP1* expression levels in the BSMV:PsAP1 group were reduced by 32%, 85%, and 55%, respectively, compared with the controls (Figure 4B). To evaluate the impact of *PsAP1* silencing on Pst pathogenicity, plants were inoculated with the Pst race CYR32 and examined after 14 days. The control leaves developed dense uredinia, whereas the BSMV:PsAP1-treated leaves exhibited a significant reduction in uredinial numbers (Figure 4C), suggesting that *PsAP1* silencing compromised pathogenicity. Furthermore, pathogen biomass quantification showed a decrease from 1.00 (control) to 0.34 in PsAP1-silenced plants (Figure 4D), further corroborating the role of *PsAP1* in the pathogenicity of Pst.

### 3.5. ROS Detection Post-Silencing

**Figure 5:**
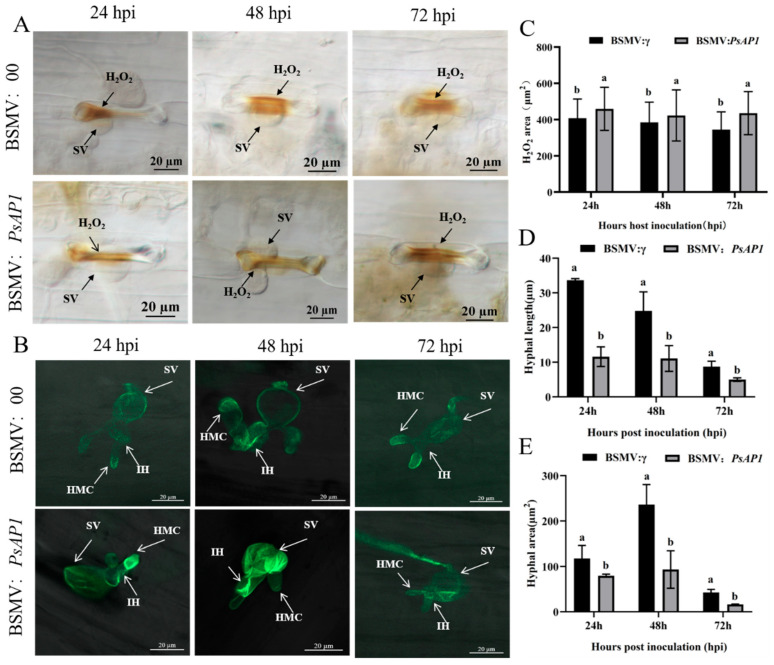
Detection of ROS accumulation and fungal development after transient silencing of *PsAP1*. (**A**) DAB staining detecting ROS accumulation (brown patches) near infection sites at 24, 48, and 72 hpi with Pst. (**B**) Fluorescence microscopic images of WGA-stained fungal structures (green) at 24, 48, and 72 hpi with Pst. SV: substomatal vesicle; IH: infection hyphae; HMC: haustorial mother cell. (**C**) Statistical analysis of areas of ROS accumulation. (**D**) Measurement of hyphal length at different time points post-inoculation. (**E**) Measurement of the hyphal area at different time points post-inoculation. Different lowercase letters at the same time point indicate significant differences (*p* < 0.05). Three biological and three technical replicates were performed for each sample.

To examine the influence of silencing on wheat immune responses against Pst and assess its effect on pathogen proliferation, histological samples were obtained from both control and BSMV:PsAP1 plants at 24, 48, and 72 hpi. Following decolorization and fixation, ROS accumulation was detected using 3,3′-diaminobenzidine (DAB) staining. The detailed experimental protocol was performed as follows: leaf segments were immersed with their morphological basal ends in 400 µL of staining solution within a 2 mL microcentrifuge tube. The samples were subsequently incubated under light conditions at a temperature maintained between 15 and 25 °C for a duration of 8 h. Upon completion of the staining reaction, the leaf tissues were subjected to decolorization using a clearing solution formulated as a 1:1 (*v*/*v*) mixture of absolute ethanol and glacial acetic acid. The decolorization process continued until the leaf segments achieved a visually transparent appearance. Notable ROS bursts were observed near infection sites in both groups (Figure 5A). Statistical analysis of ROS accumulation areas via light microscopy revealed significantly increased ROS accumulation in PsAP1-silenced plants at all time points compared to controls (Figure 5B). These findings suggest that *PsAP1* silencing enhances ROS accumulation in wheat during Pst infection.

Subsequent to the quantification of ROS, histological samples underwent heat treatment and were stained with wheat germ agglutinin (WGA) to facilitate the visualization of fungal structures. The development of hyphae was markedly inhibited at 24, 48, and 72 hpi in the silenced plants (Figure 5C). Fluorescence microscopy, with an excitation wavelength of 488 nm, was employed to assess hyphal length and area. In comparison to the control group, both the hyphal area and length were reduced in PsAP1-silenced plants at all observed time points. Specifically, hyphal length was significantly inhibited at 24 hpi, with control hyphae measuring 2.9 times longer than those in the silenced plants (Figure 5D). Furthermore, the hyphal area was significantly reduced at 48 hpi, with the control group exhibiting an area 2.53 times larger than that of the silenced group (Figure 5E).

### 3.6. Screening of Differentially Expressed Genes

**Figure 6:**
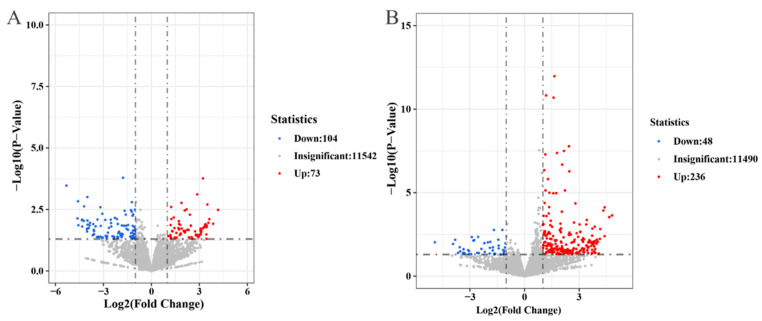
DEGs of *PsAP1* gene at 24 hpi and 48 hpi after silencing. (**A**) Volcano map of DEGs of *PsAP1* at 24 hpi after silencing. (**B**) Volcano map of DEGs of *PsAP1* at 48 hpi after silencing.

To elucidate the pathogenic mechanisms regulated by *PsAP1* in the wheat stripe rust fungus, we employed transcriptome sequencing to identify the genes and signaling pathways affected by *PsAP1*. A total of 12 cDNA libraries were constructed from samples collected at 24 and 48 hpi with Pst race CYR32. All samples exhibited GC content between 48% and 50%, with Q20 > 97% and Q30 > 92%, indicating high-quality and reliable transcriptome data. DEGs were screened using the thresholds of padj < 0.05 and |log2(fold change)| > 1. The analysis revealed 177 DEGs at 24 h, comprising 73 upregulated and 104 downregulated genes (Figure 6A), of 284 DEGs were identified at 48 h, including 236 up-regulated and 48 down-regulated genes (Figure 6B).

### 3.7. Analyses of GO and KEGG

**Figure 7:**
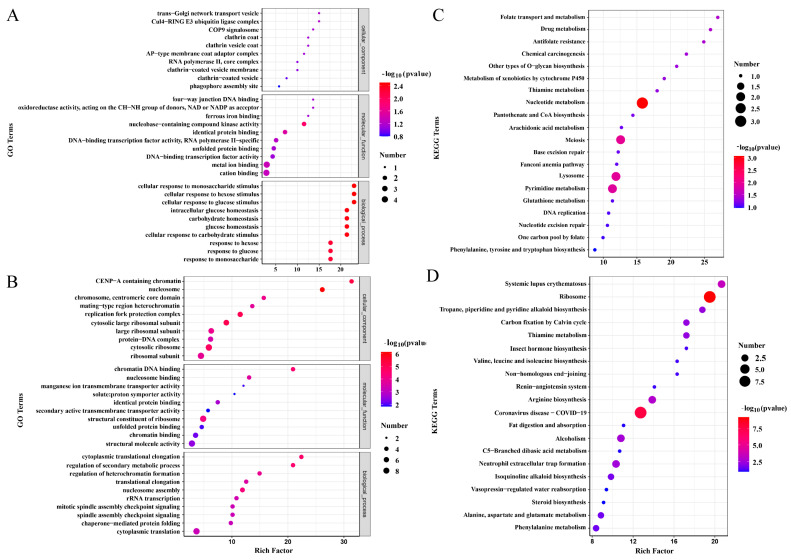
GO and KEGG enrichment analysis of DEGs after *PsAP1* silencing. (**A**) GO enrichment analysis of differentially expressed genes (DEGs) at 24 hpi. (**B**) Gene ontology (GO) enrichment analysis of DEGs at 48 hpi. (**C**) KEGG pathway enrichment analysis of DEGs at 24 hpi. (**D**) KEGG pathway enrichment analysis of DEGs at 48 hpi.

To further investigate the molecular mechanisms of *PsAP1*, we performed GO and KEGG analyses of the DEGs following *PsAP1* silencing. GO analysis revealed that at 24 h post-silencing, the DEGs were primarily localized to trans-Golgi network transport vesicles, exhibiting four-way junction DNA binding activity and participating in the cellular response to monosaccharide stimulus (Figure 7A). At 48 h post-silencing, the DEGs were mainly enriched in CENP-A-containing chromatin, demonstrating chromatin DNA-binding activity and involvement in cytoplasmic translational elongation (Figure 7B).

KEGG enrichment analysis revealed that 24 h post-silencing, DEGs were primarily enriched in signaling pathways, including folate transport and metabolism, drug metabolism, and antifolate resistance (Figure 7C). At 48 h post-silencing, the enrichment profile shifted significantly, with predominant enrichment observed in The systemic lupus erythematosus, ribosome, troponin, piperidine, and pyridine alkaloid biosynthesis pathways (Figure 7D).

GO and KEGG analyses revealed a significant temporal molecular reprogramming following *PsAP1* silencing. Early-stage changes (24 h) predominantly involve membrane transport systems and stress response mechanisms, while late-stage changes (48 h) shift towards chromatin structure and protein synthesis regulation. KEGG pathway enrichment demonstrated significant enrichment of DEGs in key metabolic and drug resistance pathways, primarily folate transport and metabolism, drug metabolism, and antifolate resistance pathways. This suggests that *PsAP1* silencing triggers core metabolic reprogramming and activates drug stress response mechanisms in the wheat stripe rust fungus. Furthermore, the enrichment of DEGs in the tropane, piperidine, and pyridine alkaloid biosynthesis pathways indicates the potentially important role of secondary metabolite synthesis and regulation in the pathogenic process of the rust fungus.

### 3.8. qRT-PCR Validation of DEGs

**Figure 8:**
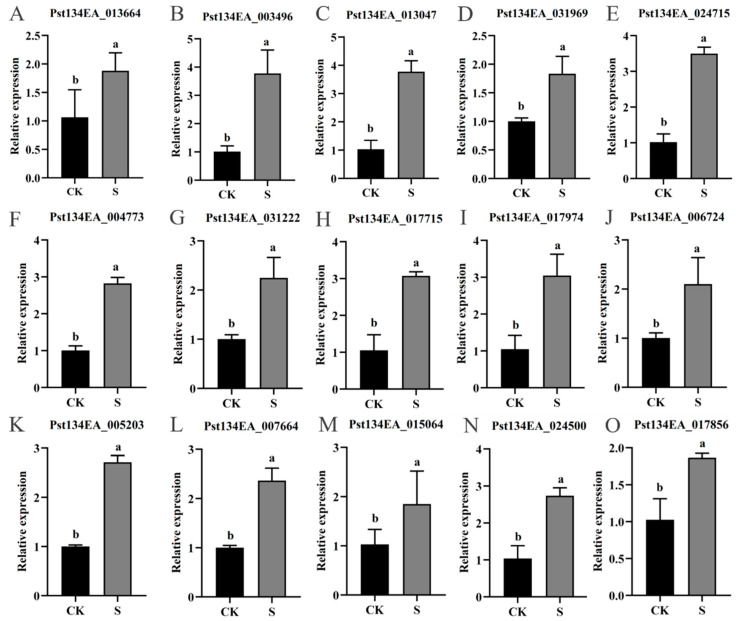
qRT-PCR validation of selected DEGs from transcriptome data. (**A**–**E**) Relative expression levels of five key DEGs at 24 hpi involved in MAPK signaling (**A**), glutathione metabolism (**B**), thiamine metabolism (**C**), and nucleotide metabolism (**D**,**E**). (**F**–**O**) Relative expression levels of ten key DEGs at 48 hpi involved in Ribosome biogenesis (**F**,**G**), MAPK signaling (**H**–**J**), Carbohydrate metabolism (**K**–**M**), thiamine metabolism (**N**), and Nitrogen metabolism (**O**). Gene IDs are shown in graphs. Different lowercase letters at the same time point indicate significant differences (*p* < 0.05). Three biological and three technical replicates were performed for each sample.

To further validate the reliability of the transcriptomic data, we validated the expression levels of five key genes from the MAPK signaling, glutathione metabolism, thiamine metabolism, and nucleotide metabolism pathways at 24 hpi using qRT-PCR. The results showed that Pst134EA_013664 (MAPK pathway) was significantly upregulated by 1.76-fold (Figure 8A); Pst134EA_003496 (glutathione metabolism) was significantly upregulated by 3.73-fold (Figure 8B); Pst134EA_013047 (thiamine metabolism) was significantly upregulated by 3.67-fold (Figure 8C); Pst134EA_031969 and Pst134EA_024715 (nucleotide metabolism) were significantly upregulated 1.83-fold and 3.43-fold, respectively (Figure 8D,E).

Similarly, the expression levels of ten key genes from the Ribosome biogenesis, MAPK signaling, Carbohydrate metabolism, thiamine metabolism, and Nitrogen metabolism pathways were validated at 48 hpi. The results showed that Pst134EA_004773 and Pst134EA_031222 (ribosome biogenesis) were significantly upregulated by 2.81-fold and 2.24-fold, respectively (Figure 8F,G); Pst134EA_017715, Pst134EA_017974, and Pst134EA_006724 (MAPK signaling) were significantly upregulated by 2.93-fold, 2.90-fold, and 2.10-fold, respectively (Figure 8H–J); Pst134EA_005203, Pst134EA_007664, and Pst134EA_015064 (carbohydrate metabolism) were significantly upregulated by 2.71-fold, 2.36-fold, and 1.80-fold, respectively (Figure 8K–M); Pst134EA_024500 (thiamine metabolism) was significantly upregulated 2.64-fold (Figure 8N); Pst134EA_017856 (nitrogen metabolism) was significantly upregulated by 1.82-fold (Figure 8O).

## 4. Discussion

Recent advances in RNA-seq have revolutionized the study of plant-pathogen interactions by enabling comprehensive transcriptional profiling. Our study leveraged this technology to investigate the role of the adaptor protein complex gene *PsAP1* in the wheat stripe rust fungus Pst. We demonstrated that *PsAP1*, which encodes a putative σ subunit of the AP-1 complex, is a critical virulence factor that orchestrates fungal development, host immunity modulation, and metabolic adaptation during infection. The functional conservation, unique subcellular localization, and multifaceted regulatory roles of PsAP1 revealed herein provide novel insights into rust fungus pathogenicity.

The early and significant upregulation of *PsAP1* during Pst infection, particularly at the substomatal vesicle and haustorial mother cell formation stages, mirrors the expression pattern of characterized effector proteins and pathogenesis-related genes in Pst [28,29,30]. This temporal expression profile strongly suggests its crucial role in establishing early infection, a notion functionally supported by the impaired pathogenicity of the *F. graminearum FgAP1* mutant in wheat coleoptiles and its partial rescue by *PsAP1* heterologous complementation. This functional complementation across fungal species underscores the conserved role of the AP-1 σ subunit in fungal pathogenesis, likely facilitating critical steps such as host penetration and biotrophic interface establishment.

Unexpectedly, PsAP1 protein localized not only to the plasma membrane but also to the nucleus in *N. benthamiana* cells. This broad localization diverges from the canonical TGN/endosome-restricted localization of AP-1 subunits in model organisms like yeast [18] and *Toxoplasma gondii* [19]. This atypical distribution suggests the potential neofunctionalization of Pst. Beyond its presumed primary role in vesicular trafficking between the TGN and endosomes, PsAP1’s nuclear presence suggests potential involvement in non-canonical functions [31]. These could include direct or indirect participation in transcriptional regulation, nuclear signal transduction, or even DNA repair or epigenetic modulation, reminiscent of some nuclear-targeted effectors from other plant pathogens. The functional implications of this nuclear localization warrant further investigation.

The critical role of *PsAP1* in Pst virulence has been directly demonstrated using BSMV-HIGS. Transient silencing of *PsAP1* led to a significant reduction in fungal sporulation, hyphal growth, and pathogen biomass coupled with an enhanced host ROS burst. This phenotype aligns with the idea that disrupting AP-1-mediated trafficking impairs the delivery of effectors and/or pathogenicity factors required for suppressing host immunity and sustaining fungal growth within plant tissue. The consistency between these findings and the loss of infectivity in the *F. graminearum FgAP1* mutant [20] reinforces the conserved importance of the AP-1 complex in fungal virulence.

Transcriptomic analysis revealed a complex regulatory network governed by PsAP1. KEGG enrichment of DEGs upon *PsAP1* silencing indicated its multifaceted involvement in critical metabolic and signaling pathways. The significant enrichment of MAPK signaling, glutathione metabolism, thiamine metabolism, and carbohydrate metabolism pathways suggested that PsAP1 orchestrates infection by coordinately regulating redox homeostasis, energy production, and biosynthetic precursor supply.

The upregulation of genes involved in glutathione metabolism highlights a potential role of *PsAP1* in mitigating host-derived ROS, possibly by ensuring the adequate transport of components essential for glutathione synthesis or function [32]. This would bolster the antioxidant capacity of the fungus and facilitate immune evasion. Furthermore, alteration of thiamine metabolism genes, particularly during haustorial development, indicates a high demand for TPP-dependent enzymes involved in central carbon metabolism. *PsAP1* might facilitate thiamine precursor acquisition or utilization, easing metabolic constraints during energy-intensive infection structure formation [33].

Moreover, the engagement of carbohydrate metabolism, especially the pentose phosphate pathway (PPP), underscores its importance in generating NADPH for redox balance and pentose phosphates for nucleotide/amino acid synthesis [34]. PsAP1 likely contributes to the efficient utilization of host-derived sugars [35,36] to fuel these processes. The temporal shift in enriched pathways from early stress responses (e.g., drug metabolism) to later processes, such as ribosome biogenesis, reflects a dynamic reprogramming of fungal physiology mediated by PsAP1 throughout infection.

In conclusion, our study suggests that PsAP1 is a central player in Pst pathogenicity. It likely functions dually by mediating the vesicular trafficking of host-derived nutrients and fungal effector proteins/cargo and by broadly influencing key metabolic and signaling pathways. This integrated role in both material transport and metabolic regulation underscores the sophistication of rust fungi in host manipulation and reveals potential vulnerabilities that could be targeted in future disease control strategies.

## 5. Conclusions

This study established *PsAP1* as a pathogenic hub in the wheat stripe rust fungus, coordinating metabolic reprogramming, ROS suppression, and vesicular trafficking to enable disease. Silencing *PsAP1* disrupts this network, cripples fungal virulence, and activates plant defense mechanisms. Thus, targeting *PsAP1* presents a promising strategy for developing transgenic wheat with broad-spectrum and durable resistance.

## Data Availability

The original contributions presented in the study are included in the article, further inquiries can be directed to the corresponding author.

## Supplementary Materials

The following supporting information can be downloaded at: https://www.mdpi.com/article/10.3390/jof12010057/s1, Figure S1: Cloning of PsAP1 gene; Table S1: Primers used in this study.
