# Supplementary material for: The AP-1 Sigma Subunit Gene PsAP1 Acts as a Key Pathogenicity Factor by Regulating Metabolic Reprogramming in Puccinia striiformis f. sp. tritici
Source: J Fungi (Basel). 2026 Jan 12;12(1):57. doi: 10.3390/jof12010057 (PMC12842939; doi:10.3390/jof12010057)
Supplement: Supplementary file 1 [file jof-12-00057-s001.zip › jof-4045860-supplementary.pdf]

Supplementary Table S1. Primers used in this study

Name	Sequences (5'-3')	Purpose
PsAP1-clone-F	ATGGCTCTCAGCTACGTCTTAC	Cloning
PsAP1-clone-R	TTATCTACCAGAGGCGATTTTTGC	
PsAP1-qrt-F	CTCAGCTACGTCTTACTCGTTTC	RT-qPCR
PsAP1-qrt-R	GTACGTCTTGCAAGCACTAATTG	
PsAP1-γ-higs-F	TTTTTAGCTAGCTGATTAATTAATGCAAGACGTACACGAATGTG	HIGS
PsAP1-γ-higs-R	TCCGTTGCTAGCTGAGCGGCCGCATCTAATTCGCATACGTTTCCGAA	
γ-higs-F	AAAGTGAGGTTAACGCAATACG	
γ-higs-R	TCAGGCATCGTTTTCAAGTT	
PsEF-F	TTCGCCGTCCGTGATATGAGACAA	
PsEF-R	ATGCGTATCATGGTGGTGGAGTGA	
TaEF-F	TGGTGTCATCAAGCCTGGTATGGT	
TaEF-R	ACTCATGGTGCATCTCAACGGACT	
PsAP1-higs-qrt-F	ATCAACTGGCAACGGAGAAT	
PsAP1-higs-qrt-R	TTGGGTGACATAGTCGTAAACC	
PsAP1-1132-dingwei-F	CGCTCTAGAACTAGTGGATCCATGGCTCTCAGCTACGTCTTACTCG	Subcellular localization
PsAP1-1132-dingwei-R	GATAAGCTTGATATCGAATTCTCTACCAGAGGCGATTTTTGCC	
1132-dingwei-F	AGATGAACTTCAGGGTCAGCTT	
1132-dingwei-R	CTATCCTTCGCAAGACCCTTCC	
Pst134EA_013664-qrt-F	CTCATTCGCCAGCTCAAGATA	Validation of Transcriptome Data
Pst134EA_013664-qrt-R	TTGGCTGGTATATCCGGTTTC	
Pst134EA_003496-qrt-F	ACTTGAGGTTCTACGGGATATTG	
Pst134EA_003496-qrt-R	CCTTGTAGAGCTTTGCGATTTG	
Pst134EA_013047-qrt-F	GTTCTTAGACGAAGTGGGTGTAG	
Pst134EA_013047-qrt-R	AGATGGTCGAGGTGAACAAAG	
Pst134EA_031969-qrt-F	TCAGGATTGGCCCAAGTTT	
Pst134EA_031969-qrt-R	ACTGCTCTCTACTGACGAAATG	
Pst134EA_024715-qrt-F	GTGCGTCCCAAGATGGATATTA	
Pst134EA_024715-qrt-R	CGGCCGTTGTGTAAGTCTAA	
Pst134EA_004773-qrt-F	TCAAGACGTTTCAGGACCAC	
Pst134EA_004773-qrt-R	GCTCGGAAAGCGCTAAATTC	
Pst134EA_031222-qrt-F	CAAGAGGGCTTCTCGAGTTATC	
Pst134EA_031222-qrt-R	ATGGCAACCTCGTCACATAC	
Pst134EA_017715-qrt-F	GGCTTCCGAGGCTTTGATTA	
Pst134EA_017715-qrt-R	CTGAGTGTCCTTGCGATAGATG	
Pst134EA_017974-qrt-F	AGGTCTTACTCGTCCCTTCT	
Pst134EA_017974-qrt-R	GGAGATGAGGTGCGGTTATATT	
Pst134EA_006724-qrt-F	TCAGCGCCTAACAACTCTTC	
Pst134EA_006724-qrt-R	CTTGGATGAGTCCTGTGTAACC	
Pst134EA_005203-qrt-F	CCCAGACCGAAAGTCGAAAT	
Pst134EA_005203-qrt-R	GAGCATGAGTGCTACAGATAGG	
Pst134EA_007664-qrt-F	ACCTCACCAGCAATCCATATAC	
Pst134EA_007664-qrt-R	TCGTTTCGAGAGTGCTTTATCC	

Pst134EA_015064-qrt-F	GGACGTTACTACAAGGCGATT	
Pst134EA_015064-qrt-R	GTTCCAGTGCGTTCATTCATTAG	
Pst134EA_024500-qrt-R	ACATCTGACTGCGGTGTAATC	
Pst134EA_017856-qrt-F	TACTACCTGCAAAGGCCAATC	
Pst134EA_017856-qrt-R	GTACGAACACTTCACCCACTC	
FgAP1-LBCK 800	ACTCCTTGATCGAGATAACGGG	
FgAP1-RBCK 800	TCTGACGACGGCCATTGC	
47-RB-F	CTGGCAAAAATCGCCTCTGGTAGATAAGGCAATGTGTGCGAGCTT	Construction of *F. graminearum* Mutant and Complementation Vector
47-LB-R	AACGAGTAAGACGTAGCTGAGAGCCATGACGATATATCGAATCAAGCC	
47-PGTN-SF	ACGAGCTGTACAAGTGAGCGGCCGCACTCCTTGATCGAGATAACGGG	
47-PGTN-SR	TTTCAGTAACGTTAAGTGCGGCCGCAGGATCGCATATGCTCTTTGAA	
47-PGTN-XF	ACGAGCTGTACAAGTGAGCGGCCGCAGATTCGAAAGTGGTTTATAGACG	
47-PGTN-XR	TTTCAGTAACGTTAAGTGCGGCCGCTCTGACGACGGCCATTGC	
PGTN-F	CTGAGCAAAGACCCCAACG	
PGTN-R	CTTATCGAGATCCTGAACACCA	

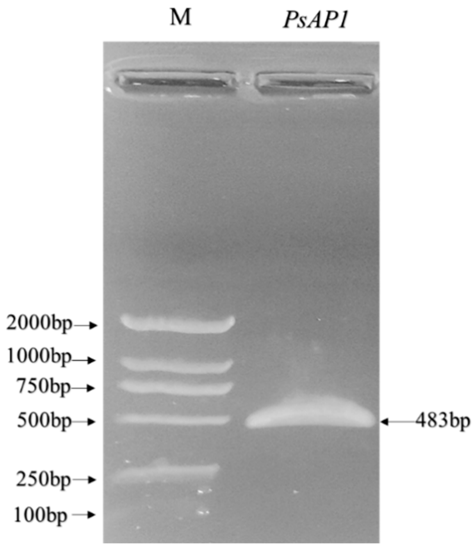

Figure. S1. Cloning of *PsAP1* gene

Note: M: 2,000 DNA Marker
